# Exercise-Induced Alternations of Adiponectin, Interleukin-8 and Indicators of Carbohydrate Metabolism in Males with Metabolic Syndrome

**DOI:** 10.3390/biom13050852

**Published:** 2023-05-18

**Authors:** Karol Makiel, Agnieszka Suder, Aneta Targosz, Marcin Maciejczyk, Alon Haim

**Affiliations:** 1 Department of Anatomy, Faculty of Physical Rehabilitation, University of Physical Education, 31-571 Cracow, Poland; 2 Department of Physiology, Faculty of Medicine, Jagiellonian University Medical College, 31-531 Cracow, Poland; 3 Department of Physiology and Biochemistry, Faculty of Physical Education and Sport, University of Physical Education, 31-571 Cracow, Poland; 4 Department of Pediatric Endocrinology and Diabetes, Faculty of Health Sciences, Ben-Gurion University of the Negev, Beer-Sheva 653, Israel; 5 Soroka University Medical Center, Beer-Sheva 151, Israel

**Keywords:** adiponectin, interleukin-8, metabolic syndrome, obesity, physical activity, HOMA-AD, HOMA-TG

## Abstract

Adiponectin (ADIPO) and interleukin-8 (IL-8) are proteins that play a significant, albeit opposing, role in metabolic syndrome (MetS). The reported data on the effect of physical activity on the levels of these hormones in the population of people with MetS are conflicting. The aim of the study was to evaluate the changes in hormone concentrations, insulin-resistance indices and body composition after two types of training. The study included 62 men with MetS (age 36.6 ± 6.9 years, body fat [BF] = 37.53 ± 4.5%), randomly assigned to: an experimental group EG1 (n = 21) with aerobic exercise intervention, an experimental group EG2 (n = 21) with combined aerobic and resistance exercise intervention, both for 12 weeks, and a control group CG (n = 20) without interventions. Anthropometric measurements and body composition (fat-free mass [FFM], gynoid body fat [GYNOID]), as well as a biochemical blood analysis (adiponectin [ADIPO], interleukin-8 [IL-8], homeostatic model assessment—adiponectin (HOMA-AD) and homeostatic model assessment—triglycerides (HOMA-TG) were performed at baseline, and at 6 and 12 weeks of intervention and 4 weeks after the intervention (follow-up). Intergroup (between groups) and intragroup (within each group) changes were statistically evaluated. In the experimental groups EG1 and EG2, no significant changes were observed in the ADIPO concentration, but a decrease of GYNOID and insulin-resistance indices was confirmed. The aerobic training led to favorable changes in IL-8 concentration. The use of combined resistance and aerobic training led to improved body composition, decreased waist circumference and better insulin-resistance indices in men with MetS.

## 1. Introduction

The criteria of metabolic syndrome (MetS) include central obesity, high blood pressure, lipid disorders and hyperglycemia [1]. MetS leads to many health consequences, including type 2 diabetes, gout [2], cardiovascular diseases (CVD), acute coronary syndrome, stroke, malignancies [3] and sleep apnea [4]. The scale of the MetS problem affects 20–25% of the adult urban population [5]. Obesity, especially central obesity, plays an important role in the development of MetS [6] and is associated with an increased risk of CVD, T2D and some cancers [7]. Each of the components of MetS can predispose patients to CVD, and the risk increases when a number of these components co-occur in one person [8,9].

The process of MetS and obesity treatment begins with lifestyle modification [10,11]. The key changes are the modification of the diet [12] and increased physical activity (PA). Insufficient levels of PA and a sedentary lifestyle are among the most important factors determining the development of MetS [13,14]. Physical exercise itself has a health-promoting effect, independent of weight loss, by acting on several mechanisms, including the inhibition of pro-inflammatory processes and the stimulation of anti-inflammatory pathways, as well as by affecting the synthesis of adipokines that regulate insulin sensitivity. High-intensity activity seems to give the best results, particularly the combination of aerobic and resistance exercises, which have achieved a significant anti-inflammatory effect in patients with type 2 diabetes and MetS [15], influencing the metabolism of the adipose tissue and skeletal muscles.

Both the adipose tissue and skeletal muscles are endocrine organs that conduct a specific dialogue, releasing cytokines, adipokines and myokines—hormones that reach their receptors, playing an important role in the homeostasis of the body [16,17]. They regulate, among other things, the energy and metabolic processes of the body [18,19]. The adipose tissue and skeletal muscles are key regulators of carbohydrate tolerance [20,21].

An example of information transferred from the adipose tissue to skeletal muscles is the production of adiponectin (ADIPO)—synthesized mainly in adipose tissue, whose Adipo1 receptors are located in the skeletal muscles. ADIPO also journeys to the Adipo2 receptor located in the liver [22]. It is responsible for fatty acids oxidation in skeletal muscles and the inhibition of glucose production in the liver, improving the energy homeostasis of the whole body. ADIPO has an anti-inflammatory function, reducing inflammation in various types of tissues [23]. Low levels of ADIPO have been observed in the population of people with MetS and abdominal obesity [24]. ADIPO has anti-atherosclerotic and insulin-sensitizing properties [25]. With respect to insulin sensitization, ADIPO has been shown to reduce blood glucose levels by inhibiting hepatic gluconeogenesis and enhancing insulin signaling in skeletal muscles [26].

As a marker of adipose tissue dysfunction, an index based on the ADIPO/LEP ratio (ADIPO/LEP ratio) has been introduced [27]. To calculate the ADIPO/LEP ratio, the concentration of circulating leptin (LEP) is used—a protein produced mainly by the adipose tissue in amounts proportional to the level of obesity. LEP is involved in the regulation of food intake, energy homeostasis and other physiological processes [28]. In obesity and MetS, the concentration of leptin increases, which is also a marker of inflammation [29]. The ADI-PO/LEP ratio is therefore a marker illustrating the pathophysiological function of both adipokines [27]. In addition, the ADIPO/LEP ratio decreases with an increase in the number of MetS risk factors [30]. An increase in the ADIPO/LEP ratio was associated in epidemiological studies with a reduced risk of atherosclerosis, as well as with a reduced risk of some types of cancer [27].

The cytokine interleukin-8 (IL-8), responsible for the increase of pro-inflammatory macrophages (M1) in adipose tissue, acts in opposition to ADIPO [31]. IL-8 is a pro-inflammatory cytokine synthesized, among others, in adipocytes, and its excessive production can lead to insulin resistance, type 2 diabetes and atherosclerosis [32,33,34]. Elevated levels of IL-8 have been observed among people with MetS [35], although there are reports indicating the opposite relationship [36]. Researchers have noted that exercise does not typically increase circulating IL-8 [37], despite evidence suggesting that IL-8 is released from skeletal muscle during exercise and acts locally [38].

Current knowledge on the impact of physical activity on the level of ADIPO, AD-IPO/LEP ratio and IL-8 does not give clear conclusions and requires more studies, preferably clinical, randomized and with a group of more than 20 people [39]. Based on the fact that physical activity is beneficial for health [40], the aim of the study was to investigate how two types of 12-week exercise training affected the parameters of body composition, ADIPO concentrations, ADIPO/LEP ratio and IL-8, as well as indicators of insulin resistance in men with MetS and how the tested parameters changed after a 4-week observation without scheduled training. We hypothesized that aerobic–resistance training would be associated with more favorable changes in hormone concentrations, i.e., an increase in ADIPO and a decrease in IL-8, with a decrease of insulin-resistance indices and an improvement in body composition, i.e., an increase in fat-free mass and a decrease in body fat and waist circumference, compared to aerobic training.

## 2. Materials and Methods

### 2.1. Study Design

The study was designed as a randomized, prospective controlled study. A detailed description of the research methods was presented in previous papers [41,42]. The aim of this study was to compare the 12-week effect of two types of physical training on ADIPO and IL-8 levels and carbohydrate metabolism indices in men with metabolic syndrome (MetS), compared to men with MetS not undertaking physical activity (control group CG). The interventions involved applying aerobic training (EG1) and training combining resistance and aerobic exercise (EG2). Body composition parameters and selected indicators of the MetS were used to monitor changes. After 12 weeks of intervention, a period of 4 weeks of observation without scheduled training took place, in which the participants of the groups themselves decided on the number of training sessions or lack thereof.

The process of assigning to groups was carried out randomly; each of the study participants chose an opaque envelope with the group number. During the statistical analysis of the results and the performance of the biochemical determinations, the staff were unaware of the group assignment. Due to the form of the intervention or its absence, no blind trial was used.

The study involved 62 Caucasian men aged 30 to 45 (mean age 36.6 ± 6.9) who met the main selection criterion, concerning an increased waist circumference (WC) above 94 cm (which is one of the criteria for the diagnosis of MetS) and two of the other four MetS criteria for men: systolic (SBP) ≥ 130 mmHg or diastolic (DBP) ≥ 85 mmHg; HDL C < 1.03 mmol/L; triglycerides > 1.7 mmol/L; fasting plasma GL ≥ 5.6 mmol/L or drug treatment for the disorder presented (International Diabetes Federation, IDF) [43].

Participants were randomly assigned to 3 groups:

1. Experimental group: EG1 of men (age: 34.21 ± 6.06) with MetS (n = 21) performing aerobic exercise (BMI: 34.57 ± 4.58; BF: 38.03 ± 4.82);
2. Experimental group: EG2 of men (age: 37.37 ± 7.08) with MetS (n = 21) performing combined aerobic–resistance exercise (BMI: 33.14 ± 4.32; BF: 37.33 ± 4.30);
3. Control group: CG of men (age: 38.26 ± 7.43) with MetS (n = 20) who did not engage in any physical activity (BMI: 33.20 ± 4.31; BF: 37.22 ± 4.37).

There were no differences between age and basic somatic parameters before the interventions.

Apart from being male and meeting the MetS diagnosis, the following criteria were included in the study: age 30–45, medical certificate of no contraindications to undertake aerobic–resistance health training, and written consent for voluntary participation in the research project.

The exclusion criteria for the research project comprised: medical contraindications to resistance and aerobic training, too-low attendance at trainings in intervention groups (minimum attendance above 90%) and others, thoroughly presented in a previous paper [41].

The volunteers underwent training and received a written description of the objectives, procedures and the planned course of the research project. Each of the participants could withdraw from the study at any time without any consequences. During the project, there were situations leading to a reduction in the number of study participants. The main exclusion factor was absence during control measurements—9 participants. As a result of introducing excessive changes in diet (alcohol intake), 2 participants were excluded; as a result of infectious diseases, 3 participants were excluded; and as a result of too-low attendance during training, 3 patients were excluded (<90% attendance).

**Figure 1:**
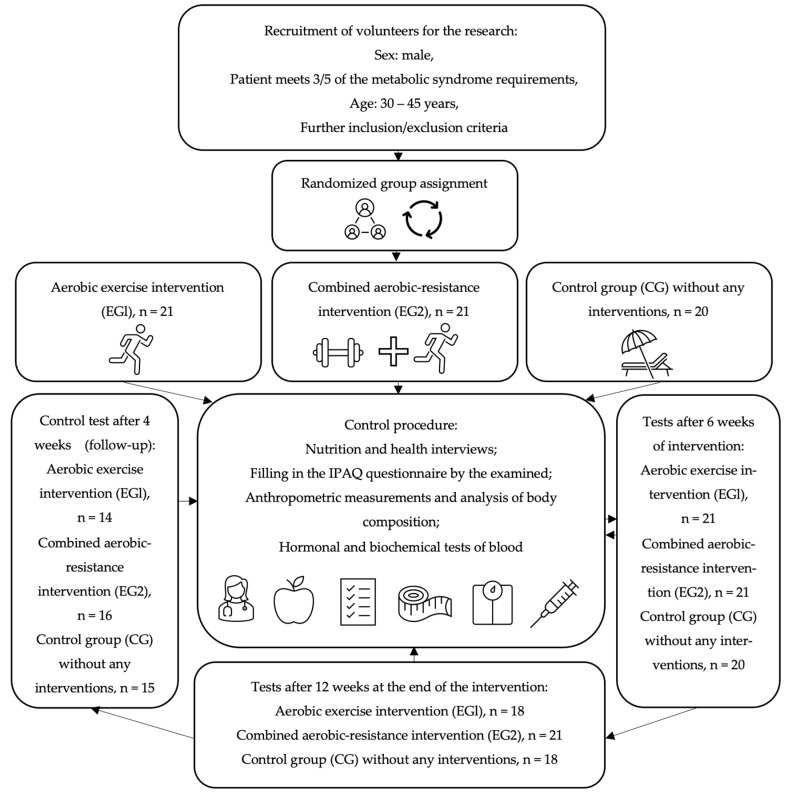
The flowchart of the study.

All subjects were trained by the same personal coach and asked not to change their diet, not to undertake physical activity other than with a trainer, and to maintain their regimen of medications and dietary supplements during the observation. All participants of the study gave written consent to the processing of personal data, voluntary participation in the study and the use of the obtained results for scientific purposes. The research project was approved by the Ethics Committee of the District Medical Chamber in Krakow (90/KBL/OK/2020). The studies were registered in the register of clinical trials on the ANZCTR (Australian New Zealand Clinical Trials Registry) platform: ACTRN 12622001394730. The flowchart of the study is presented in Figure 1.

### 2.2. Methods

The research project took 16 weeks, during which the evaluation was carried out 4 times: before the intervention, after 6 weeks of training, after 12 weeks of training and after 16 weeks of the project—the last 4 weeks was the period of observation without scheduled training. The following parameters were assessed during the control weeks.

#### 2.2.1. Anthropometry

Body mass (BM) [kg], body height (BH) [cm] and waist circumference (WC) [cm] were used in the study. BM, BH and WC were measured in a standing position, in underwear, with the head in the Frankfurt plane. BM was measured with a medical scale (Beurer PS 240, Budapest, Hungary) with an accuracy of 50 g. BH was measured with an accuracy of 1 mm with a stadiometer (Seca 231 stadiometer, Hamburg, Germany). Waist circumference (WC) was measured last during free exhalation using an anthropometric tape, between the upper edge of the iliac crest and the lower edge of the costal arch. Based on the obtained BM and BH, the body mass index (BMI) [kg/m^2^] was calculated.

#### 2.2.2. Body Composition

Dual-Energy X-ray Absorptiometry (DEXA) was applied to assess body composition: fat-free mass (FFM) [%], body fat (BF) [%] and gynoid body fat (GYNOID) [%]. Evaluation of body composition was carried out with the Lunar Prodigy Primo PR+352163 (Chicago, IL, USA) device according to the manufacturer’s guidelines.

#### 2.2.3. Hormones

Fasting blood samples were collected after a one day break from workout, in the morning, from the basilic, cephalic or median cubital vein into test tubes (Vacumed^®^ system, F.L. Medical, Torreglia, Italy) by experienced nurses. The collected blood was centrifuged (RCF 1.000× *g*) immediately after collection for 15 min at 4 °C (MPW-351R, MPW Med. Instruments, Warsaw, Poland) and serum was collected and stored at −80 °C until further study (BIO Memory 690L, Froilabo, Paris, France).

The concentrations of adiponectin (ADIPO), leptin (LEP) and interleukin-8 (IL-8) were measured using commercially available ELISA kits according to the manufacturer’s protocol. The human Adiponectin ELISA Kit (catalogue number E09) was purchased from Mediagnost (Reut-Lingen, Germany). The human Leptin Sandwich ELISA Kit (catalogue number EIA-2395) was purchased from DRG Instruments GmbH (Marburg, Germany). The IL-8 ELISA kit (catalogue number EIA-4700) was purchased from DRG Instruments GmbH (Marburg, Germany). An ELx 808 spectrophotometric microplate reader (BioTek, Winooski, VT, USA) was used to determine the optical density at 450 nm. Marking was performed in the Laboratory of Genetics and Molecular Biology at the Department of Physiology, Jagiellonian University Medical College, Cracow, Poland.

Index of adiponectin-to-leptin ratio (ADIPO/LEP ratio) was calculated based on the formula:

ADIPO/LEP ratio = ADIPO (µg/mL)/LEP (µg/mL)

#### 2.2.4. Biochemical Blood Indices

Fasting plasma glucose (FPG) [mmol/L] was determined via the enzymatic method using a Cobas c701/702 biochemical analyzer (Roche Diagnostics International Ltd., Mannheim, Germany). Serum insulin concentration (INS) [µIU/mL] was determined via the electrochemiluminescence method (ECLIA) using the Cobas e801 apparatus (Roche Diagnostics International Ltd., Mannheim, Germany). The determinations were performed in accordance with the manufacturer’s instructions using reagents dedicated to the GLUC3 and Elecsys Insulin analyzers, respectively.

Using the specifications of the Architect ci-4100 clinical chemistry analyzer (Abbott Laboratories), serum triglyceride (TG) [mg/dl] levels were determined via spectrophotometry.

Evaluation of sensitivity to insulin was performed with the use of the homeostatic model assessment—adiponectin (HOMA-AD) [44] and homeostatic model assessment—triglycerides (HOMA-TG) [45], calculated based on the formula:

HOMA-AD = INS (µU/mL) × FPG (mmol/L)/ADIPO (µg/mL);

HOMA-TG = INS (µU/mL) × FPG (mmol/L)/TG (mg/dL)

#### 2.2.5. Evaluation of Total Energy Expenditure and Energy Value of Diet

The International Physical Activity Questionnaire (IPAQ) [46] was employed to evaluate the daily energy expenditures. The total energy expenditure (TEE), measured in kilocalories per day, was computed as the sum of non-exercise activity thermogenesis (NEAT) assessed through the IPAQ questionnaire and the energy expenditures associated with the interventions implemented in the EG1 and EG2 groups.

To evaluate the energy value of the participants’ diets, a clinical dietician conducted a 24 h nutrition interview using the nutrition record method. The data were analyzed using the DietaPro program (version 4.0, Institute of Food and Nutrition, Warsaw, Poland) to quantitatively assess the nutrition habits and monitor any changes in the diet during the intervention. Based on the obtained results, a report of dietary nutrients was generated: proteins [g], carbohydrates [g] and fats [g].

### 2.3. Exercise Interventions

The exercise interventions were conducted at a fitness club and supervised by a personal coach. The training sessions were carried out at the same time of day (evening, 6–9 pm) by the same personal coach, in a room with consistent temperature (22 degrees Celsius) and humidity. Adherence to the intervention was monitored using a session attendance checklist, and participants who dropped out from more than 10% of the training sessions for 12 weeks were excluded from the analysis.

Individualized planning and monitoring of aerobic and resistance training intensity were based on the guidelines of the American College of Sports Medicine [47]. The One Repetition Maximum (1 RM) was determined before resistance training (Supplement). The load and number of repetitions were recorded and converted into 1 RM based on the 1 RM calculator using Brzycki’s formula [48,49].

The intervention aimed to achieve 3 training sessions per week, which resulted in 3 × 5.5 MET for a week equivalent to resistance training, and 3 × 6 MET for running [50].

#### 2.3.1. Aerobic Training

The aerobic training intervention (Figure S1, Supplement) involved three sessions per week in groups of up to five participants at a fitness club. The training started with a five-minute warm-up on a treadmill (Technogym New Excite Run Now 500, Cesena, Italy) at 50% of maximal heart rate (HR max). HR max was calculated based on the formula: 208 − 0.7 × age (years) [51]. Heart rate during training was monitored using the Polar M200 GPS Running Watch with Wrist-Based Heart Monitor (Kempele, Finland).

Next, the participants increased the intensity of their workout to 70% HR max by adjusting their velocity or angle on the treadmill, resistance on the upright bikes (Technogym Artis, Cesena, Italy) or range of motion or resistance on the x-trainer (Precor EFX556i Elipsa, Woodinville, WA, USA). The aerobic exercises mainly consisted of fast walking or jogging on the treadmill; however, in the case of reporting pain from the musculoskeletal system, the participants had an option to change the device. The training was continuous and maintained a steady HR, with a duration of 45 min. Following the aerobic training, participants stretched the muscle groups they had engaged for 10 min.

#### 2.3.2. Combined Aerobic–Resistance Training

The aerobic–resistance intervention (Table S1, Supplement) was conducted three times per week in groups of up to five participants under the supervision of a personal coach. One session of exercises lasted 60 min. The training started with a 5 min aerobic warm-up on a treadmill to reach an intensity of 50% HRmax.

The initial resistance training comprised three complex exercises involving the whole body, such as one-arm dumbbell row, squats and push-ups, with four sets and 120 s breaks between them. Due to the body’s adaptation to training, in the second week of intervention, the resistance training procedure was changed to push–pull and the training volume was changed to 3 sets of 6 exercises with 90 s breaks. After 3 weeks of intervention, the training was performed in 3 series of 9 exercises with 60 s breaks. The load was gradually increased from the first week, from 50% 1RM to 70% 1RM in the second and the remaining 10 weeks of intervention [52]. The progression of load of the resistance exercises during the intervention and follow-up for EG2 was statistically significant in the analyzed period (Table S2, Supplement).

After resistance exercises, there was an aerobic training element: the participants trained with an intensity of 50% HR max in the first week and 70% HR max from the second week of intervention on a treadmill (Technogym New Excite Run Now 500, Cesena, Italy), upright bike (Technogym Artis, Cesena, Italy) or x-trainer (Precor EFX556i Elipsa, Woodinville, WA, USA). To avoid overloading the joints of the lower extremities, the subjects could use these three devices alternately.

The duration of the resistance training sessions was 30, 35 and 40 min, respectively, followed by 20, 15 and 10 min of aerobic training, respectively. The training session ended with the stretching phase (5 min).

### 2.4. Statistical Analysis

The Shapiro–Wilk test was used to examine the distribution of the variables being analyzed. To compare the effects of an intervention on changes in the analyzed variables in the experimental groups and control group, the one-way ANOVA test with repeated measures and post hoc comparison (Tukey’s test) was employed. Homogeneity of variance within the groups was tested with Levene’s test.

The size effect (ES) for the ANOVA test was calculated using the *ƞ*^2^ coefficient, which is the ratio of the sum of squares (*SS*) for the effect to the total sum of squares (*SS*). The squared eta coefficient interpretation follows Cohen’s guidelines: 0.1 ≤ 0.3 (low effect), 0.3 ≤ 0.5 (moderate effect) and ≥0.5 (high effect) [53].


$$\eta^{2}=\frac{{SS}_{effect}}{{SS}_{total}}$$

Pearson’s correlation coefficient (r) was used to calculate correlations between LEP, IL-8 and HOMA-AD and other measured parameters. The interpretation of the Pearson correlation in the range < 0–1 > was made as follows: 0 ≤ r < 0.3, no or very weak correlation; 0.3 ≤ r < 0.5, moderate correlation; 0.5 ≤ r < 0.7, strong correlation; 0.7 ≤ r ≤ 1, very strong correlation [54].

To explain the variation in ADIPO concentrations, multiple regression was employed, utilizing an econometric linear multiple regression model assessed by the least-squares method. In the model, the residual standard errors and test *p*-values were corrected using robust standard errors corrected for heteroscedasticity.

The number of participants required to demonstrate statistical significance was based on previously published studies in the field. Probability of error (α) 0.05, power (1 − β) 0.80 and mean effect size (d) 0.8 were used to calculate the sample size and the tested sample was n = 54.

In all the analyses, effects were considered significant if their probability value *p* was less than the assumed significance level α = 0.05 (*p* < 0.05). The ggplot2 package in the RStudio IDE of the R programming language was applied to perform all calculations.

## 3. Results

After performing health training intervention both in EG1 (*p* = 0.02) and EG2 (*p* = 0.01), a significant increase of total energy expenditure (TEE) [kcal/day] was confirmed between the initial measurements and measurements in the 6^th^ (*p* < 0.001) week of intervention (Table 1). In the 12th week of intervention, the significance level of EG1 was *p* < 0.001 and EG2 was *p* = 0.04. In follow-up, an increase of TEE was also confirmed in EG1 (*p* < 0.001) and in EG2 (*p* = 0.03). No significant changes were found in TEE in CG. A significant difference in TEE between the intervention groups and the CG in the 6th week of observation was confirmed (*p* = 0.03).

When analyzing the balance of nutrients during the study, no changes in the level of protein supplied in the diet in the EG1 group were confirmed (Table 1). In EG2, changes in the level of supplied proteins were observed (*p* < 0.001), but a visible increase in consumption occurred only at the follow-up stage (*p* = 0.01). CG confirmed the variability in protein intake between measurements (*p* = 0.04).

In the EG1 and EG2 intervention groups, carbohydrate consumption increased between measurements (*p* = 0.04) (Table 1). In both groups, the highest intake was confirmed in the follow-up period (*p* = 0.01).

Significant changes in consumption also occurred in the case of fats supplied in the diet (Table 1). Changes between measurements in EG1 (*p* = 0.03) and CG (*p* = 0.01) were confirmed. The increase in fat consumption was noticeable in the EG2 group after 6 (*p* = 0.04), 12 (*p* = 0.03) and 16 (*p* = 0.01) weeks compared to the measurement before the intervention. Similar relationships were observed in the CG group; despite the lack of intervention, fat consumption increased in the 6th (*p* = 0.03), 12th (*p* < 0.001) and 16th (*p* = 0.04) weeks of observation (Table 1).

Analyzing the body composition of the people participating in the study, the most beneficial changes regarding FFM increase, GYNOID and WC decrease were confirmed in the EG2 group (Table 2). No changes in FFM were confirmed in the case of the EG1 and CG groups, while in the EG2 group there was a significant increase in FFM between measurements (*p* < 0.001); after 16 weeks, the observed increase was 5.8% (*p* < 0.001).

In the case of GYNOID, it decreased after 6 weeks of intervention in the EG1 group (*p* = 0.03) (Table 2). A significant change between measurements occurred in the EG2 group (*p* < 0.001), in which decreases in GYNOID levels were confirmed after 6 (*p* = 0.02) and 16 (*p* < 0.001) weeks. No significant changes were observed in the CG group.

After the WC analysis, changes in EG1 and CG were not confirmed (Table 2). However, in the EG2 group, changes were found both between measurements (*p* < 0.001) and in each of the analyzed measurement moments (*p* = 0.01). The decrease in WC was 3.8 cm after 12 weeks of intervention (*p* = 0.01).

By analyzing changes in insulin resistance indices, changes between measurements (*p* = 0.02) in HOMA-AD values in the EG2 group were confirmed (Table 3). There was an increase after 6 weeks (*p* = 0.03), followed by a decrease in HOMA-AD in subsequent measurements. No significant changes in HOMA-AD values were observed in the EG1 and CG groups.

In the case of the HOMA-TG index, changes between measurements in the EG1 (*p* = 0.04) and EG2 (*p* = 0.03) intervention groups were confirmed (Table 3). The greatest changes were observed in the EG2 group, where after 16 weeks the decrease was 39% (*p* = 0.04). There were no significant changes in the CG.

Observations of ADIPO fluctuations did not confirm significant changes in the concentration of the analyzed hormone both within groups and between groups (Table 4). However, changes in the ADIPO/LEP ratio in the EG1 group were found between the measurements (*p* < 0.001) and after 6 (*p* = 0.01) and 16 (*p* < 0.001) weeks.

**Figure 2:**
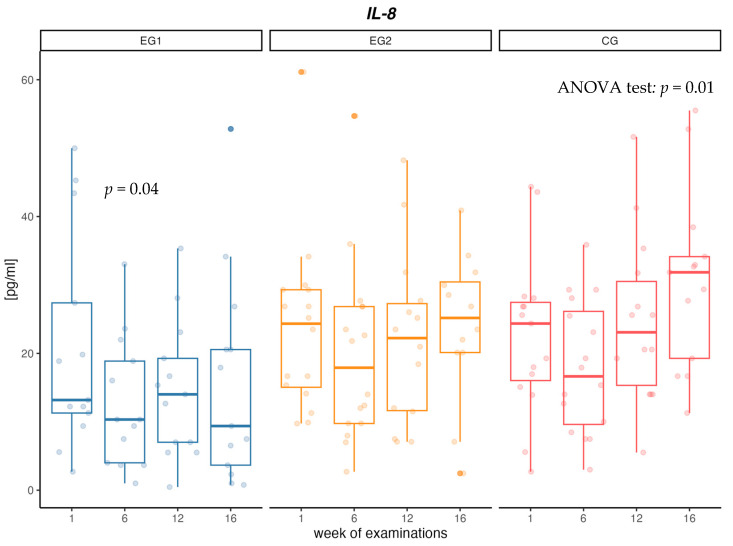
Changes in interleukin-8 (IL-8) concentration [pg/mL] in aerobic group (EG1), aerobic–resistance group (EG2) and control group (CG) during weeks of examinations.

When analyzing changes in the concentration of IL-8 cytokine, no changes in concentrations between measurements in the EG1 and EG2 intervention groups were confirmed. In the EG1 group, there was a decrease in the concentration of IL-8 in the first 6 weeks of the intervention (*p* = 0.04). In contrast, in CG there was a significant increase in IL-8 concentration (*p* = 0.01) between measurements of 36% over 16 weeks. Additionally, at week 16, a significant difference in IL-8 concentration between CG and EG1 was confirmed (*p* = 0.03) (Table 4, Figure 2).

Significant correlations (Table S3, Supplement) were confirmed in EG1 between ADIPO and dietary carbohydrate (r = −0.27), dietary fat (r = −0.35), GYNOID (r = 0.33) and insulin resistance indices HOMA-AD (r = −0.63) and HOMA-TG (r = −0.48). No significant correlations were observed for IL-8. There were significant correlations between HOMA-AD and the level of carbohydrates (r = 0.37), fats (r = 0.34), WC (r = 0.36) and HOMA-TG (r = 0.81), and negative significant correlations for HOMA-AD and ADIPO (r = −0.63). Analyzing the correlations in the EG2 group, significant correlations between ADIPO and carbohydrates (r = 0.43), GYNOID (r = 0.42), HOMA-AD (r = −0.56) and ADIPO/LEP ratio (r = 0.68) were confirmed. For IL-8, one significant correlation with HOMA-AD was observed (r = −0.28). In the case of HOMA-AD, there were significant correlations with GYNOID (r = −0.27), WC (r = 0.39), HOMA-TG (r = 0.55), ADIPO (r = −0.56), ADIPO/LEP ratio (r = −0.50) and IL-8 (r = −0.28). In CG, significant correlations were observed between ADIPO and TEE (r = 0.26), HOMA-AD (r = −0.59) and HOMA-TG (r = −0.29). In the case of IL-8, the correlation with TEE was confirmed (r = 0.43). HOMA-AD had a significant linear relationship with HOMA-TG (r = 0.53) and ADIPO (r = −0.59).

The applied multiple regression model demonstrated that both HOMA-AD and GYNOID were significantly connected with the concentration of ADIPO (*p* < 0.001). The variability of ADIPO was explained by the analyzed variables in 36% (value of R^2^ model = 0.36) (Table 5).

## 4. Discussion

The aim of the study was to compare the 12-week effect of two types of physical training on ADIPO and IL-8 concentrations and carbohydrate metabolism indices in men with MetS compared to men with MetS not undertaking physical activity, and to evaluate changes in these parameters after 4 weeks of observation without scheduled training. Training interventions over 12 weeks did not change the concentration of ADIPO, but significant correlations were observed between ADIPO and HOMA-AD and GYNOID. The applied multiple regression model showed that both variables explained 36% of ADIPO variability. Aerobic exercise was associated with a decrease in IL-8 concentration after 6 weeks of intervention in men with MetS. The use of a combined resistance and aerobic training led to a significant increase in FFM, a decrease in GYNOID and WC and a reduction in the level of insulin resistance in the group of men with MetS.

In a meta-analysis of studies on people with pre-diabetes or diabetes, in which the participants were overweight or obese and often had MetS, it was observed that physical exercise increased the concentration of ADIPO. Furthermore, it was emphasized that the results in improving the concentration of ADIPO were observed in studies using aerobic exercise, whereas other forms of physical exercise did not bring such results [55]. Similar results were presented in earlier meta-analyses [39,56]. Additionally, Balducci et al. [15] showed that aerobic exercise, but also a combination of resistance and aerobic exercise, bring beneficial changes in the concentration of ADIPO (increase by 36% and 38%) and in the level of insulin resistance in patients with MetS, despite the lack of changes in body mass and the level of adipose tissue. In our study, however, no changes in ADIPO concentration were observed in any of the analyzed groups. The authors suggest that significant changes in the concentration of ADIPO are influenced by a higher negative energy balance caused by aerobic exercise compared to other types of activity [57]. There are also reports in which the interventions did not have a significant effect on the concentration of ADIPO. In five clinical trials evaluating the effect of a 10% reduction in body mass on the concentration and expression of ADIPO in plasma, no significant changes were observed [58,59,60,61,62]. The authors noticed that in a short time (up to 12 weeks), the concentration of the hormone increased—hence, favorable changes in the level of ADIPO were observed, after which the fluctuations usually stabilized. Moreover, one study has shown that a reduction of 5 to 10% of body mass has little or no effect on the concentration of ADIPO [63].

Our study confirmed changes in the level of GYNOID, achieving a significant reduction after 6 weeks in both intervention groups. We also observed a correlation between ADIPO and GYNOID in the intervention groups, which may suggest that while achieving greater beneficial changes in body composition, the concentration of ADIPO could increase to obtain significant differences. Moreover, there have been reports that ADIPO is negatively correlated with the level of android and total adipose tissue and positively correlated with insulin sensitivity [64].

Our study confirmed significant correlations between ADIPO and two indices of insulin resistance: HOMA-AD and HOMA-TG. Khan et al. [45] proposed HOMA-TG as a good indicator for the diagnosis of MetS, indicating that HOMA-TG may provide better diagnostic performance in the diagnosis of MetS than HOMA-IR, HOMA2 and QUICKI. Matsuhisa et al. [44] noted that HOMA-AD showed a greater correlation with the level of insulin resistance than HOMA-IR. However, the concentration of ADIPO is used to calculate HOMA-AD, which in turn increases the correlation between the described hormone and the index of insulin resistance. Our research also showed that both indicators were strongly correlated with each other and that the values of both indicators changed significantly after the aerobic–resistance intervention: after 16 weeks, the decrease in HOMA-AD was 46%, while HOMA-TG was 39%. Such beneficial changes in carbohydrate metabolism, resulting from the use of a combination of resistance and aerobic training, may offer perspectives in the treatment of people with MetS, insulin resistance and type 2 diabetes.

The processes that can occur under the influence of training sessions should be analyzed in detail in order to properly understand the relation between ADIPO, insulin resistance and physical activity in males with MetS and obesity. Skeletal muscles are the main area of carbohydrate metabolism in the human body; moreover, they are also the main area of insulin resistance development [65]. Chronic positive energy balance of the body, leading to obesity, results not only in disorders at adipokine levels but also in accumulation of adipose tissue in the liver and skeletal muscles, and subsequently in improper metabolic response, including mainly insulin resistance. ADIPO, after connecting to its receptor in muscles (ADIPO R1) through An Adaptor Protein 1 (APPL1), affects the activation of many signaling pathways, including the insulin receptor substrate (IRS) pathway, AMP-activated protein kinase (AMPK) and p38 mitogen-activated protein kinase (p38 MAPK), leading to the regulation of blood glucose. The main insulin-regulating mechanism affected by ADIPO is the IRS, whose functioning is impaired in obesity [66].

In obesity, transcription factors such as SERBP1c may lead to the development of lipotoxicity in skeletal muscles through the deposition of triglycerides, acyl-CoA, phosphatides, diacylglycerols (DAG) and ceramides [67]. In our research, in the EG2 group, there was a significant increase in FFM in the first 6 weeks of the intervention, amounting to 4.1%, and in the same period the level of GYNOID decreased. However, HOMA-AD and HOMA-TG insulin resistance indicators increased by 31.6% and 22.5%. The main component affecting the increase in insulin resistance was the increase in insulin concentration in the analyzed period, which was shown in our former work [41]. The increase in insulin concentration may have resulted from a limited ability to respond to insulin at its receptor, caused by lipotoxicity related to the insulin receptor substrate (IRS) or a limitation in the function of glucose transporter type 4 (GLUT4) [68,69]. Insulin resistance could also be associated with the occurrence of muscle microdamages, caused mainly by eccentric contractions, the prolonged phase of which is characteristic of resistance training, affecting the reduction of GLUT4 levels and partial inability to resynthesis of glycogen [70]. The probability of occurrence of muscle microdamage was higher in this group due to the initial adaptation processes as a result of increasing the volume and intensity of training.

Our results showed that the ADIPO/LEP ratio decreased significantly after 6 weeks of aerobic intervention. The occurring relation resulted from a significant increase of LEP level, presented in our previous work [42] and the lack of significant changes in the level of ADIPO during the analyzed period. LEP can react to the changes taking place in the population of people with MetS faster than ADIPO. Such biological fluctuations of the described hormones were also confirmed [63]. Frühbeck et al. [27] indicated that the ADIPO/LEP ratio is characterized by a higher level of correlation with insulin resistance than either ADIPO or LEP alone; however, such a relationship was not confirmed in our research.

In our study, a significant 35% decrease in IL-8 concentration was observed after the first 6 weeks of intervention in the group using aerobic training. Decreased concentration of IL-8, in relation to the initial concentration, was observed until the end of the research project in the group with aerobic training, but no such dependencies were found in the group with aerobic–resistance training. The results presented in the meta-analysis show that the physical activity of people with MetS leads to a decrease in the concentration of IL-8 [71]. However, the results are still conflicting, since same studies did not observe changes in serum concentrations of IL-8. In a large population study of 489 MetS people, men and women over 55 years of age who engaged in moderate or intense exercise of a minimum of 150 min per week, no significant changes in IL-8 levels were observed during one year of follow-up [72]. In their paper, Guo et al. showed that increased plasma cytokine levels (TNF-α, IL-6 and IL-8) were associated with reduced strength gain during resistance training [73].

In our study, in the group without physical activity, a gradual increase in IL-8 concentration between measurements was observed and the cytokine level was 45% higher in the control group compared to the group with aerobic intervention. According to paper Bruun et al., [74] high levels of IL-8 are secreted from human adipose tissue, and the accumulation of IL-8 in this tissue may be partly responsible for the increase in circulating concentrations of IL-8 seen in obese individuals. Elevated levels of IL-8 secreted from myotubes in diabetes create a muscle microenvironment that stimulates reduced capillarity in diabetes, ultimately limiting the availability of substrates including glucose, exacerbating impaired muscle glucose clearance and contributing to the diabetes phenotype [75]. Failure to treat obesity and metabolic disorders leads to a further increase in inflammation in the body and numerous health complications [76].

Currently, the physiological function of IL-8 in skeletal muscle is still unknown; thus, further research is needed to identify the potential of IL-8 as a diagnostic biomarker.

The current study has some limitations. The participants of the project increased their nutrient intake despite recommendations to maintain their current diet. Determination of specialized biochemical indicators, e.g., the level of glycated hemoglobin (HbA1c) could allow for a better determination of the level of insulin resistance and give the possibility of a more precise description of the correlation and assessment of modified HOMA indices.

In conclusion, undertaking a 12-week aerobic–resistance training program, despite the lack of significant changes in the level of ADIPO, led to a decrease in insulin resistance expressed as HOMA-AD and HOMA-TG. In the aerobic training group, no significant changes were observed in the ADIPO concentration, but a decrease in insulin resistance expressed in HOMA-TG was confirmed. The level of ADIPO was significantly related to the level of GYNOID and HOMA-AD. Under the influence of aerobic–resistance training, there was a significant increase in FFM and a decrease in GYNOID and WC. Aerobic training led to a decrease in IL-8 after 6 weeks of intervention. The use of aerobic training, as well as a combination of aerobic and resistance training, brought health benefits to men with MetS. In our study, a combination of aerobic and resistance training resulted in more benefits. More tests are recommended in order to select the correct training method in the treatment of MetS.

## Figures and Tables

**Table 1:** Total energy expenditure (TEE) and diet nutrients: proteins, carbohydrates and fats in the aerobic group (EG1), aerobic–resistance group (EG2) and control group (CG).

	Group	Week 1 Baseline	Week 6 Intervention	Week 12 Intervention	Week 16 Follow-Up	*p*-Value			
		$\bar{𝑿}$ ± SD	$\bar{𝑿}$ ± SD	$\bar{𝑿}$ ± SD	$\bar{𝑿}$ ± SD	Test ANOVA (ES)	d 6-1 (ES)	d 12-1 (ES)	d 16-1 (ES)
TEE [kcal/day]	EG1	597.46 ± 195.93	823.37 ± 175.76	835.18 ± 234.05	838.00 ± 350.75	0.02 (0.15)	<0.001 (−3.28)	<0.001 (−1.37)	<0.001 (0.69)
	EG2	553.84 ± 85.63	735.17 ± 119.64	797.89 ± 383.25	749.17 ± 430.71	0.01 (0.12)	<0.001 (−2.01)	0.04 (−0.67)	0.03 (−0.50)
	CG	634.06 ± 221.38	627.97 ± 197.18	652.36 ± 186.92	690.23 ± 205.14	0.56 (0.00)	0.87 (−0.05)	0.77 (−0.09)	0.18 (−0.41)
	*p*-value	0.45	0.03 *	0.20	0.53				
Proteins [g]	EG1	125.50 ± 53.37	126.50 ± 39.20	120.93 ± 29.63	134.93 ± 44.13	0.46 (0.02)	0.92 (−0.03)	0.63 (0.13)	0.18 (−0.38)
	EG2	144.06 ± 34.09	137.31 ± 25.45	125.00 ± 21.06	154.67 ± 40.64	<0.001 (0.11)	0.34 (0.29)	0.05 (0.63)	0.01 (−0.91)
	CG	148.80 ± 39.74	135.06 ± 21.90	126.14 ± 19.87	152.85 ± 35.07	0.04 (0.11)	0.18 (0.42)	0.07 (0.57)	0.86 (−0.05)
	*p*-value	0.32	0.57	0.83	0.38				
Carbo -hydrates [g]	EG1	295.79 ± 67.74	294.29 ± 68.92	310.29 ± 70.32	346.21 ± 81.50	0.04 (0.08)	0.95 (0.02)	0.47 (−0.20)	0.01 (−0.87)
	EG2	304.38 ± 79.41	319.00 ± 70.14	338.00 ± 69.59	356.83 ± 81.90	0.04 (0.03)	0.60 (-0.15)	0.04 (−0.70)	0.01 (−1.01)
	CG	316.20 ± 65.40	304.81 ± 73.44	320.21 ± 50.77	347.69 ± 77.61	0.16 (0.03)	0.22 (0.37)	0.59 (0.16)	0.24 (−0.36)
	*p*-value	0.74	0.63	0.52	0.94				
Fats [g]	EG1	110.43 ± 43.78	104.43 ± 29.79	122.21 ± 34.85	117.79 ± 40.99	0.03 (0.03)	0.43 (0.22)	0.05 (−0.57)	0.13 (−0.43)
	EG2	92.38 ± 22.99	103.44 ± 18.23	106.57 ± 18.30	103.25 ± 22.73	0.08 (0.07)	0.04 (−0.67)	0.03 (−0.73)	0.01 (−0.94)
	CG	99.20 ± 25.82	113.56 ± 21.63	121.57 ± 19.96	113.38 ± 26.22	0.01 (0.23)	0.03 (−0.72)	<0.001 (−1.09)	0.04 (−0.69)
	*p*-value	0.30	0.42	0.20	0.50				

* post hoc EG1-CG: *p* = 0.03; d 6-1, d 12-1, d 16-1—differences in results obtained after 6 and 12 weeks of interventions, respectively, and after 4 weeks of follow-up in relation to measurements taken before interventions, $\bar{𝑿}$—mean, SD—standard deviation, *p* < 0.05—statistically significant difference, ES—effect size.

**Table 2:** Body composition: fat-free mass (FFM), gynoid body fat (GYNOID) and waist circumference (WC) in the aerobic group (EG1), aerobic–resistance group (EG2) and control group (CG).

	Group	Week 1 Baseline	Week 6 Intervention	Week 12 Intervention	Week 16 Follow-Up	*p*-Value			
		$\bar{𝑿}$± SD	$\bar{𝑿}$ ± SD	$\bar{𝑿}$ ± SD	$\bar{𝑿}$ ± SD	Test ANOVA (ES)	d 6-1 (ES)	d 12-1 (ES)	d 16-1 (ES)
FFM [%]	EG1	63.09 ± 4.81	63.74 ± 5.04	63.85 ± 5.26	64.56 ± 4.95	0.13 (0.00)	0.07 (−0.58)	0.05 (−0.64)	0.07 (−0.58)
	EG2	62.56 ± 5.23	65.14 ± 5.44	65.63 ± 4.69	66.17 ± 4.30	<0.001 (0.06)	<0.001 (−1.60)	<0.001 (−1.32)	<0.001 (−1.82)
	CG	59.23 ± 16.97	54.90 ± 22.00	62.51 ± 4.96	61.57 ± 4.80	0.19 (0.13)	0.17 (0.45)	0.50 (0.21)	0.14 (0.49)
	*p*-value	0.58	0.11	0.28	0.07				
GYNOID [%]	EG1	37.36 ± 5.57	36.21 ± 5.85	36.74 ± 6.33	36.04 ± 5.10	0.07 (0.01)	0.03 (0.68)	0.23 (0.35)	0.05 (0.59)
	EG2	35.91 ± 4.88	34.49 ± 4.62	35.08 ± 4.36	33.84 ± 4.60	<0.001 (0.04)	0.02 (0.95)	0.24 (0.39)	0.00 (1.59)
	CG	35.91 ± 6.22	35.90 ± 6.62	36.18 ± 5.99	36.24 ± 6.84	0.16 (0.00)	0.42 (0.29)	0.15 (−0.57)	0.51 (−0.23)
	*p*-value	0.73	0.68	0.75	0.53				
WC [cm]	EG1	114.7 ± 10.93	113.8 ± 12.01	114.0 ± 12.87	113.7 ± 12.56	0.53 (0.02)	0.14 (0.43)	0.44 (0.21)	0.25 (0.32)
	EG2	114.8 ± 11.64	113.2 ± 11.55	111.0 ± 10.33	111.3 ± 11.08	<0.001 (0.00)	0.01 (1.10)	0.01 (1.15)	0.01 (1.15)
	CG	115.3 ± 10.54	117.4 ± 11.22	119.1 ± 11.09	119.3 ± 12.26	0.50 (0.05)	0.49 (−0.25)	0.07 (−0.57)	0.21 (−0.24)
	*p*-value	0.99	0.55	0.18	0.24				

EG1—aerobic group, EG2—aerobic–resistance group, CG—control group, d 6-1, d 12-1, d 16-1—differences in results obtained after 6 and 12 weeks of interventions, respectively, and after 4 weeks of follow-up in relation to measurements taken before interventions, $\bar{𝑿}$—mean, SD—standard deviation, *p* < 0.05—statistically significant difference, ES—effect size.

**Table 3:** Concentrations of homeostatic model assessment—adiponectin (HOMA-(AD) and homeostatic model assessment—triglycerides (HOMA-TG) in the participants’ blood in the aerobic group (EG1), aerobic–resistance group (EG2) and control group (CG).

	Group	Week 1 Baseline	Week 6 Intervention	Week 12 Intervention	Week 16 Follow-Up	*p*-Value			
		$\bar{𝑿}$ ± SD	$\bar{𝑿}$ ± SD	$\bar{𝑿}$ ± SD	$\bar{𝑿}$ ± SD	Test ANOVA (ES)	d 6-1 (ES)	d 12-1 (ES)	d 16-1 (ES)
HOMA-AD	EG1	624.63 ± 733.61	548.62 ± 840.29	676.14 ± 899.51	487.19 ± 740.30	0.78 (0.00)	0.87 (0.05)	0.83 (0.07)	0.52 (0.21)
	EG2	509.01 ± 425.69	669.88 ± 534.83	403.09 ± 353.73	277.22 ± 199.89	0.02 (0.30)	0.03 (0.71)	0.43 (0.30)	0.57 (0.22)
	CG	748.10 ± 789.61	572.24 ± 443.26	1085.25 ± 1362.91	704.11 ± 802.73	0.26 (0.11)	0.83 (0.07)	0.20 (−0.46)	0.81 (−0.08)
	*p*-value	0.61	0.85	0.19	0.36				
HOMA-TG	EG1	2.80 ± 1.43	2.33 ± 1.73	2.41 ± 1.27	2.26 ± 1.23	0.04 (0.04)	0.11 (0.55)	0.05 (1.15)	0.07 (0.66)
	EG2	2.27 ± 1.18	2.78 ± 2.16	1.53 ± 0.48	1.38 ± 0.49	0.03 (0.17)	0.77 (−0.13)	0.06 (0.70)	0.04 (0.73)
	CG	2.99 ± 2.53	2.91 ± 2.12	2.59 ± 1.47	3.09 ± 2.77	0.97 (0.00)	0.78 (0.10)	0.91 (−0.04)	0.96 (−0.02)
	*p*-value	0.52	0.75	0.05	0.10				

EG1—aerobic group, EG2—aerobic–resistance group, CG—control group, d 6-1, d 12-1, d 16-1—differences in results obtained after 6 and 12 weeks of interventions, respectively, and after 4 weeks of follow-up in relation to measurements taken before interventions, $\bar{𝑿}$—mean, SD—standard deviation, *p* < 0.05—statistically significant difference, ES—effect size.

**Table 4:** Concentrations of adiponectin (ADIPO), leptin-to-adiponectin ratio (ADIPO/LEP ratio) and interleukin-8 (IL-8) in participants’ blood plasma in the aerobic group (EG1), aerobic–resistance group (EG2) and control group (CG).

	Group	Week 1 Baseline	Week 6 Intervention	Week 12 Intervention	Week 16 Follow-Up		*p*-Value		
		$\bar{𝑿}$ ± SD	$\bar{𝑿}$ ± SD	$\bar{𝑿}$ ± SD	$\bar{𝑿}$ ± SD	Test ANOVA (ES)	d 6-1 (ES)	d 12-1 (ES)	d 16-1 (ES)
ADIPO [ng/mL]	EG1	4.61 ± 2.01	4.04 ± 1.84	4.29 ± 1.67	4.39 ± 2.01	0.32 (0.01)	0.08 (0.51)	0.32 (0.28)	0.52 (0.18)
	EG2	4.57 ± 2.82	3.98 ± 2.26	3.85 ± 2.13	4.00 ± 1.95	0.32 (0.02)	0.14 (0.47)	0.32 (0.30)	0.53 (0.20)
	CG	4.36 ± 1.87	4.83 ± 2.29	3.77 ± 2.24	4.43 ± 2.19	0.30 0.03	0.09 (−0.54)	0.51 (0.20)	0.23 (−0.37)
	*p*-value	0.95	0.47	0.77	0.85				
ADIPO/LEP ratio	EG1	2.77 ± 4.86	0.71 ± 0.79	4.08 ± 7.60	0.68 ± 0.87	<0.001 (0.45)	0.01 (0.70)	0.95 (0.03)	<0.001 (0.73)
	EG2	0.73 ± 0.76	0.70 ± 0.71	0.68 ± 0.55	0.93 ± 0.86	0.40 (0.01)	0.87 (0.05)	0.40 (0.26)	0.43 (−0.25)
	CG	3.21 ± 6.79	0.52 ± 0.47	0.77 ± 0.83	0.73 ± 0.47	0.16 (0.13)	0.15 (0.43)	0.20 (0.39)	0.38 (0.27)
	*p*-value	0.31	0.66	0.08	0.71				
IL-8 [pg/mL]	EG1	20.87 ± 15.80	13.64 ± 9.34	15.79 ± 9.43	16.91 ± 15.42	0.37 (0.06)	0.04 (0.63)	0.13 (0.45)	0.46 (0.21)
	EG2	23.75 ± 12.74	21.73 ± 13.87	22.83 ± 12.93	24.15 ± 11.39	0.39 (0.03)	0.45 (0.22)	0.54 (0.19)	0.34 (−0.29)
	CG	22.63 ± 11.67	17.92 ± 9.70	24.71 ± 12.23	30.71 ± 13.22	0.01 (0.14)	0.08 (0.56)	0.61 (−0.15)	0.10 (−0.52)
	*p*-value	0.85	0.22	0.08	0.02 *				

* post hoc EG1-CG: *p* = 0.02; EG1—aerobic group, EG2—aerobic–resistance group, CG—control group, d 6-1, d 12-1, d 16-1—differences in results obtained after 6 and 12 weeks of interventions, respectively, and after 4 weeks of follow-up in relation to measurements taken before interventions, $\bar{𝑿}$—mean, SD—standard deviation, *p* < 0.05—statistically significant difference, ES—effect size.

**Table 5:** Parameters of multiple regression model of the adiponectin (ADIPO) dependent variable.

Dependent Variable	Parameter Assessment	Standard Error	t Value	*p*-Value
Free parameter	1.47	0.87	1.69	0.09
HOMA-AD	−0.002	0.0002	−8.43	<0.001
GYNOID	0.10	0.02	4.41	<0.001

Free parameter—intercept, HOMA-AD—homeostatic model assessment—adiponectin, GYNOID—gynoid body fat.

## Data Availability

Data are available on request from the corresponding author.

## Supplementary Materials

The following supporting information can be downloaded at: https://www.mdpi.com/article/10.3390/biom13050852/s1, Figure S1: A comprehensive strategy for the aerobic program intended for a group engaged in aerobic activities (EG1); Table S1: A comprehensive strategy for a combined aerobic and resistance training program intended for a group engaged in aerobic–resistance activities (EG2); Table S2: The progressions of loads [kg] in selected resistance exercises during the intervention and follow-up in comparison to baseline in the aerobic–resistance group EG2; Table S3: The value of the Pearson correlation for variables in the aerobic group (EG1), aerobic–resistance group (EG2), and control group (CG).
