# Supplementary material for: Exercise-Induced Alternations of Adiponectin, Interleukin-8 and Indicators of Carbohydrate Metabolism in Males with Metabolic Syndrome
Source: Biomolecules. 2023 May 18;13(5):852. doi: 10.3390/biom13050852 (PMC10216579; doi:10.3390/biom13050852)
Supplement: Supplementary file 1 [file biomolecules-13-00852-s001.zip › biomolecules-2373517-supplementary.pdf]

biomolecules

MDPI

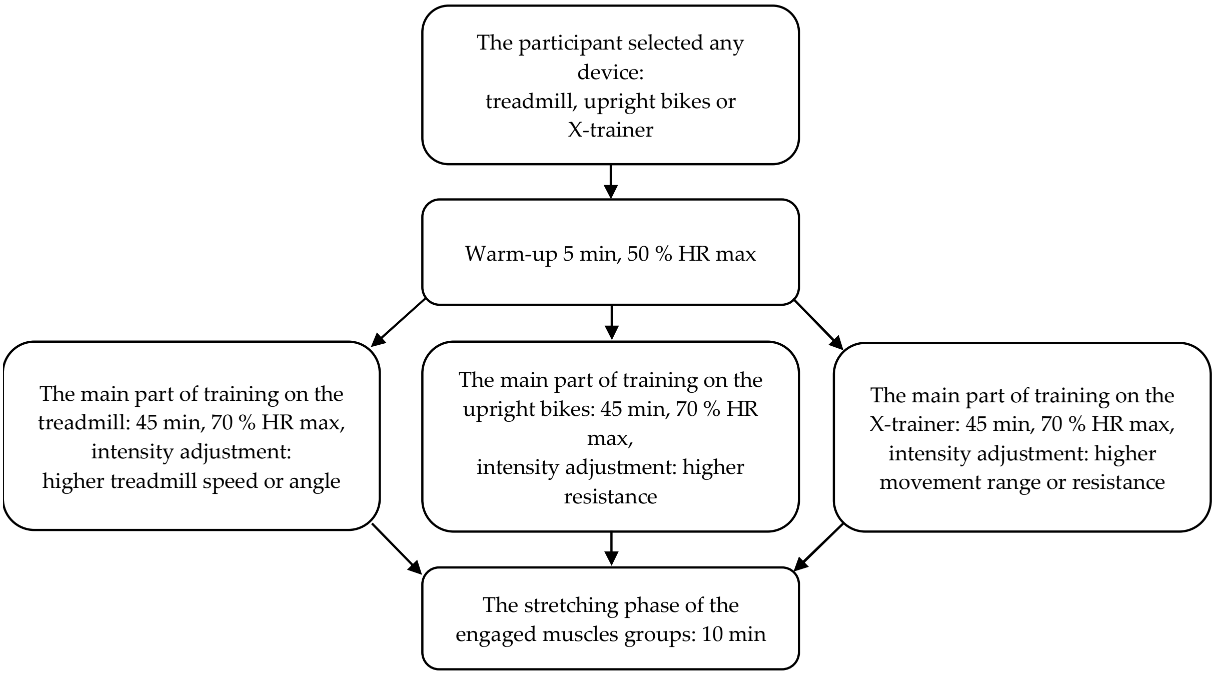

HR max – maximal heart rate

Figure S1. A comprehensive strategy for the aerobic program intended for a group engaged in aerobic activities (EG1).

Table S1. A comprehensive strategy for a combined aerobic and resistance training program intended for a group engaged in aerobic–resistance activities (EG2).

	**Training Sessions 1 - 3**	**Training Sessions 4 - 6**	**Training Sessions 6 <**
Type of training	Whole-body training	Training of antagonistic parts	Training of antagonistic parts
Volume of resistance training [exercises x series x repetitions]	3 x 4 x 15	6 x 3 x 12	9 x 3 x 12
Intensity of resistance training [% 1 RM]	50	70	70
Breaks between series [min]	2	1.5	1
Duration of resistance training [min]	30	35	40
Duration of aerobic training [min]	20	15	10
Intensity of aerobic training [% HR max]	50	70	70
Specialised exercises	One-arm row with dumbbell<br>Supported push-ups (Smith machine)<br>Supported sit-ups (with bar)<br>Front support (plank)	Standing dumbbell press<br>Barbell bench press<br>Reverse grip lat pulldown<br>Hip thrust lying<br>Bent dumbbell row	Dumbbell deadlift<br>Cable tricep extension<br>Standing dumbbell curl

HR max—maximal heart rate, 1RM—one repetition maximum.

Table S2. The progressions of loads [kg] in selected resistance exercises during the intervention and follow-up in comparison to baseline in the aerobic–resistance group EG2.

Time of observation	Barbell Bench press [kg]	Lat Pull Down [kg]	Dumbbell Squat [kg]
Baseline	63.36 ± 12.92	11.58 ± 2.56	44.51 ± 8.26
After 6 weeks of intervention	72.78 ± 14.77	13.15 ± 2.84	51.77 ± 9.84
After 12 weeks of intervention	76.65 ± 15.04	14.89 ± 2.64	56.78 ± 9.77
After 16 weeks, follow-up period	79.32 ± 17.29	14.91 ± 2.02	57.74 ± 10.78
p- value	0.00	0.00	0.00

p-value—ANOVA test.

Table S3. The value of the Pearson correlation for variables in the aerobic group (EG1), aerobic–resistance group (EG2), and control group (CG).

	TEE[1] [kcal/day]	Proteins[1] [g]	Carbohydrates[1] [g]	Fats[1] [g]	FFM[1] [%]	GYNOID[1] [%]	WC[1] [cm]	HOMA-AD[1]	HOMA-TG[1]	ADIPO[1] [ng/ml]	ADIPO/LEP ratio[1]	IL-8[1] [pg/ml]
ADIPO EG1 [ng/ml]	−0.23	−0.14	−0.27*	−0.35*	−0.24	0.33*	0.03	−0.63*	−0.48*	1.00	0.23	0.03
ADIPO EG2 [ng/ml]	0.14	−0.08	−0.43*	0.06	−0.13	0.42*	−0.16	−0.56*	−0.04	1.00	0.68*	0.12
ADIPO CG [ng/ml]	0.26*	0.17	0.05	−0.04	−0.06	0.23	−0.02	−0.59*	−0.29*	1.00	0.00	0.11
IL-8 EG1 [ng/ml]	−0.16	0.11	−0.07	−0.01	−0.23	0.20	0.19	−0.19	−0.22	0.03	0.02	1.00
IL−8 EG2 [ng/ml]	−0.26	0.18	−0.07	−0.01	0.04	0.24	−0.12	−0.28*	−0.09	0.12	−0.06	1.00
IL-8 CG [ng/ml]	0.43*	−0.10	0.01	−0.05	0.23	−0.25	−0.19	−0.02	0.14	0.11	−0.08	1.00
HOMA-AD EG1	0.26	0.04	0.37*	0.34*	−0.19	0.02	0.36*	1.00	0.81*	−0.63*	−0.19	−0.19
HOMA-AD EG2	−0.05	0.05	0.21	0.14	−0.14	−0.27*	0.39*	1.00	0.55*	−0.56*	−0.50*	−0.28*
HOMA-AD CG	0.04	−0.20	−0.09	−0.02	0.08	−0.11	0.11	1.00	0.53*	−0.59*	−0.08	−0.02

*—statistically significant $p < 0.05$; ADIPO EG1—concentrations of adiponectin in EG1 taken from the four timepoints; ADIPO EG2—concentrations of adiponectin in EG2 taken from the four timepoints; ADIPO CG—concentrations of adiponectin in CG taken from the four timepoints; IL-8 EG1—concentrations of interleukin-8 in EG1 taken from the four timepoints; IL-8 EG2—concentrations of interleukin-8 in EG2 taken from the four timepoints; IL-8 CG—concentrations of interleukin-8 CG taken from the four timepoints, total energy expenditure (TEE), fat-free mass (FFM), gynoid body fat (GYNOID), waist circumference (WC), homeostatic model assessment—adiponectin (HOMA-AD), homeostatic model assessment—triglycerides (HOMA-TG), adiponectin (ADIPO), adiponectin-to-leptin ratio (ADIPO/LEP ratio), interleukin-8 (IL-8), 1—taken from the four timepoints, corresponding to the group and measurement week in column 1.

The course of 1RM:

The examined participants underwent the 1 RM test before the examination, and after 6, 12 and 16 weeks.

The personal coach carried out the warm-up on the treadmill (Technogym New Excite Run Now 500, Cesena, Italy) for 5 min at 60% HR. The subjects warmed up in 2 series of 10 repetitions using about 50% of their 1 RM estimated load before the beginning of the test protocol.

After a 5 min break, the subjects were instructed to perform the selected test exercise until unable to continue the exercise series while maintaining the proper technique (failure).

For the 1RM bench press test, the subjects were instructed to maintain 5-point body contact (i.e., head, back and hips with the bench, and both feet with the floor) during the test; the barbell had to touch the chest when lowered.

In the 1RM squat test, subjects were instructed to move from a standing position to a position of 90 degrees of flexion at the knee joints.

The pull-down test was performed on a training atlas. The repetition was passed when the subject made a full extension of the arms during the eccentric phase and touching the bar to the chest during the concentric phase.

A qualified personal coach controlled the range of motion to verify the correctness of the test.

The last repetition of a series occurred when the participant could not continue the exercise while maintaining the proper technique.

The obtained load and number of repetitions were converted into 1 RM based on the 1 RM calculator [49], applying the Brzycki formula [48].

References

48. Brzycki, M. Strength Testing—Predicting a One-Rep Max from Reps-to-Fatigue. J. Phys. Educ. Recreat. Dance 1993, 64, 88–90. https://doi.org/10.01080/07303084.1993.10606684.

49. Grgic, J.; Lazinica, B.; Schoenfeld, B.J.; Pedisic, Z. Test-Retest Reliability of the One-Repetition Maximum (1RM) Strength Assessment: A Systematic Review. Sport. Med. Open. 2020, 6, 31. https://doi.org/10.1186/s40798-020-00260-z.
